# Development of a predictive model for visual outcomes and risk factors of bilateral involvement for non-arteritic anterior ischemic optic neuropathy (NAION) in Chinese patients

**DOI:** 10.1186/s40662-026-00504-1

**Published:** 2026-08-14

**Authors:** Xintong Xu, Mingming Sun, Shanshan Cao, Chunyan Pan, Yuhang Wang, Biyue Chen, Andrew G. Lee, Jost Jonas, Quangang Xu, Shihui Wei, Huanfen Zhou

**Affiliations:** 1 Senior Department of Ophthalmology, Chinese PLA General Hospital, Beijing, 100853 China; 2 Department of Ophthalmology, Beijing Anzhen Hospital, Capital Medical University, Beijing, China; 3 UTMB (Galveston, TX, USA) Department of Ophthalmology, Texas A&M University, Houston, TX USA; 4 Department of Ophthalmology, Blanton Eye Institute, Houston Methodist Hospital, Houston, TX USA; 5 Departments of Ophthalmology, Neurology, and Neurosurgery, Weill Cornell Medicine, New York, NY USA; 6 Department of Ophthalmology, University of Texas MD Anderson Cancer Center, Houston, TX USA; 7 Department of Ophthalmology, The University of Iowa Hospitals and Clinics, Iowa City, IA USA; 8 Rothschild Foundation Hospital, Paris, France, 25-29 Rue Manin, 75019 Paris, France; 9 Singapore Eye Research Institute, Singapore National Eye Center, Singapore, Singapore

**Keywords:** Non-arteritic anterior ischemic optic neuropathy, Risk factors, Visual prognosis

## Abstract

**Background:**

This study aimed to explore the risk factors associated with bilateral involvement and to develop a predictive model for visual prognosis in Chinese patients with non-arteritic anterior ischemic optic neuropathy (NAION).

**Methods:**

In this retrospective database-related meta-analysis, we retrospectively identified patients with NAION from the Chinese Neuro-Ophthalmic Diseases project database between September 2020 and December 2024. The clinical characteristics of the patients were summarized. Logistic and stepwise regression analyses were performed to identify the factors associated with bilateral involvement. Patients were randomly divided into training (75%) and validation (25%) cohorts. Least absolute shrinkage and selection operator (LASSO) and logistic regression analyses were used to develop a model for predicting factors associated with a best-corrected visual acuity (BCVA) ≤ 0.3 (decimal).

**Results:**

The study included 895 patients (560 [62.57%] males, 1,128 eyes; mean age: 55.6 ± 10.4 years). Bilateral NAION was present in 226 (25.3%) patients. At the follow-up examination, 522 patients (730 eyes) had a BCVA ≤ 0.3 in 369 (50.6%) eyes. Bilateral NAION involvement was associated with hyperlipidemia (t = 2.02, *P* = 0.04), hematologic disorders (t = 2.31, *P* = 0.02), and smoking (t = 4.79, *P* < 0.01). Risk factors for a BCVA ≤ 0.3 at follow-up were a lower BCVA at onset (odds ratio [OR] = 0.72, 95% confidence interval [CI]: 0.64–0.82, *P* < 0.01), diabetes mellitus (OR = 2.97, 95% CI: 1.10–8.03, *P* = 0.03), hyperhomocysteinemia (OR = 11.59, 95% CI: 1.87–71.97, *P* = 0.01), and macular serous detachment (OR = 6.70, 95% CI: 1.34–33.40, *P* = 0.02). The area under the receiver operating characteristic curve was 0.87 (95% CI: 0.83–0.87) for the training cohort and 0.86 (95% CI: 0.80–0.86) for the validation cohort.

**Conclusions:**

Hyperlipidemia, hematologic disorders, and smoking were significant risk factors for bilateral NAION, whereas the severity of vision loss at onset, diabetes mellitus, hyperhomocysteinemia, and macular serous detachment were associated with poor visual prognosis in Chinese patients with NAION.

## Background

Non-arteritic anterior ischemic optic neuropathy (NAION) is the most common optic neuropathy in adults over 50 years, although it also occurs in younger individuals. Hypoperfusion of the posterior ciliary arteries and intrinsic vascular risk factors have been reported to contribute to the pathogenesis of NAION. NAION typically presents as an acute unilateral painless vision loss with a relative afferent pupillary defect and sectorial or diffuse edema of the optic nerve head [1, 2 ]. The proposed risk factors for NAION include systemic vascular risk factors (e.g., arterial hypertension, diabetes mellitus, and hyperlipidemia) and anatomical structural abnormalities of the optic nerve head, particularly a small optic disc. In addition, certain medications have been implicated as potential precipitants of NAION. The most frequently reported drug classes include phosphodiesterase-5 (PDE-5) inhibitors such as sildenafil, tadalafil, vardenafil, and avanafil, which are used for erectile dysfunction [3, 4 ].

Previous studies have found that the probability of developing NAION in the fellow eye within 5 years after a unilateral occurrence of NAION is approximately 15%–30% [5]. Studies involving relatively small cohorts have suggested that the primary risk factors for eventual contralateral involvement include diabetes, acute severe arterial hypotension, extraocular surgery, and drug intake [6–8], while the factors affecting visual prognosis include the initial visual acuity at the onset of NAION, an older age, diabetes, and high arterial blood pressure [9, 10 ]. Most previous studies have focused on cohorts of Western populations, and only a few investigations have been conducted in China, most of which included relatively small study sizes. Therefore, we conducted this study in a relatively large cohort of Chinese patients with NAION to evaluate the clinical characteristics of NAION in Chinese patients, develop a predictive model for assessing the future risk of NAION occurrence in the second unaffected eye, and estimate the visual prognosis.

## Methods

We collected clinical information of patients diagnosed with NAION from the Chinese Neuro-Ophthalmic Diseases Project Database (registration number: 00376–2020), a bidirectional observational national multicenter cohort study. We retrospectively identified patients with NAION that was diagnosed or confirmed by a neuro-ophthalmologist at multiple nationwide tertiary referral hospitals between September 2020 and December 2024. The inclusion criteria for NAION included the following: (1) acute monocular painless visual loss; (2) presence of a relative afferent pupillary defect; (3) sectorial or diffuse optic disc edema; and (4) arcuate or altitudinal visual field defects. The exclusion criteria were the presence of optic nerve disease or retinopathy with inflammatory, hereditary, traumatic, or other causes. Patients with arteritic anterior ischemic optic neuropathy were excluded based on clinical manifestations, auxiliary examinations, and laboratory results. The diagnosis was based on expert interpretation of the results of biochemical markers of inflammation, including platelet counts, C-reactive protein levels, and erythrocyte sedimentation rates; temporal artery biopsies showing granulomatous inflammation with or without the presence of giant cells in the internal elastic lamina; and (18)F-fluorodeoxyglucose positron emission tomography (FDG-PET) and computed tomography (CT) imaging showing vasculitis. Informed consent was obtained from all patients. The study protocol was approved by the Ethics Committee of the First Medical Center of the Chinese PLA General Hospital (S2021-127-01).

We selected risk factors that may contribute to the onset of NAION and indicators associated with poor visual prognosis in previous studies. The following clinical data were collected: sex, age, body mass index (BMI), smoking history, alcohol consumption history (i.e., regular intake of alcoholic beverages of ≥ 200 mL per week for at least 6 consecutive months prior to NAION onset), history of high-altitude living, and presence of systemic diseases including diabetes mellitus, arterial hypertension, hyperlipidemia (including cholesterol, low-density lipoprotein, and triglyceride levels), hematologic disorders, coronary artery disease (CAD), cerebrovascular disease (CVD; history of cerebrovascular accident or transient ischemic attack), cardiac arrhythmias, moderate-to-severe obstructive sleep apnea syndrome (OSAS), and nocturnal arterial hypotension diagnosed based on 24 h ambulatory blood pressure monitoring (ABPM) showing a mean nocturnal systolic blood pressure < 100 mmHg or diastolic blood pressure < 60 mmHg. A history of high-altitude living was defined as a self‑reported history of continuous residence at an altitude ≥ 2,500 m above sea level for ≥ 6 consecutive months at any point in the patient’s life. Patients were considered to have a positive history of high-altitude living regardless of whether they were still living at high altitude at the time of NAION diagnosis (i.e., both current and past residents were included). Hematologic disorders were defined as the presence of any of the following: anemia (hemoglobin level < 13 g/dL in males or < 12 g/dL in females), polycythemia (hematocrit level > 52% in males or > 48% in females), or a documented hypercoagulable state (e.g., antiphospholipid syndrome or protein C or S deficiency). Ophthalmic examination data included measurements of the best-corrected visual acuity (BCVA) at disease onset, BCVA at the last follow-up (≥ 3 months), ocular symptoms at disease onset, and fundus examination for the presence of hemorrhage, macular edema, and subretinal fluid within 1 month of NAION onset. BCVA at disease onset was measured using a standard Snellen chart at the first visit and recorded as decimal acuity; all measurements were obtained within 7 days of disease onset. Nocturnal blood pressure measurements, when available, were conducted using 24 h ABPMs [11]. A crowded optic disc (disc-at-risk) was assessed in the contralateral (unaffected) eyes of patients with unilateral NAION and was defined as an optic disc diameter < 1.5 mm and a cup-to-disc ratio < 0.2, as evaluated by fundus examination and/or optical coherence tomography (OCT) imaging of the optic nerve head. For patients who developed bilateral NAION, the disc appearance prior to second-eye involvement was used, when available.

We performed a univariate logistic regression analysis to predict the risk factors for bilateral eye involvement in the entire cohort. Subsequently, factors with *P* < 0.1 were included in the multivariate stepwise regression analysis. The unilateral or bilateral status was defined at the last documented follow-up.

After excluding patients with unavailable follow-up BCVA examination results, the data of the remaining patients were used to develop a visual prognosis model. The patient cohort was divided into two groups using simple randomization. The training cohort consisted of 75% of the patients and was used to develop the model for predicting a BCVA ≤ 0.3 (decimal) at follow-up. The validation cohort comprised 25% of the patients and was used to validate the model. Least absolute shrinkage and selection operator (LASSO) regression analysis was used to screen the factors that were associated with visual progression, and a logistic regression analysis was performed to establish the model for predicting the visual progression (BCVA ≤ 0.3 or BCVA > 0.3). In addition, multivariate logistic regression with cluster-robust standard errors (patient-level clustering) was performed to account for within-subject correlations in both eyes of the same patient. The receiver operating characteristic (ROC) curve and the area under the ROC curve (AUC) were used to assess the ability of the model to estimate the occurrence of a BCVA ≤ 0.3 at follow-up. Non-parametric bootstrapping with 1,000 resamples was performed, and calibration curves were created to assess the agreement between the actual observations. The model was applied to the validation cohort to further assess its stability using the AUC values and calibration curves.

Statistical analyses were conducted using the SPSS (version 25.0; IBM Corporation, Chicago, IL, USA) and R (version 4.3.1; R Foundation for Statistical Computing, Vienna, Austria) software. Graphs were generated using the R software. Continuous variables are presented as mean and standard deviation or as median and interquartile range (IQR), while categorical variables are summarized by frequencies and percentages. The Student's t-test and Pearson's chi-square test were used to compare baseline characteristics between the two cohorts for continuous variables and proportions, respectively. Statistical significance was set at *P* < 0.05.

## Results

### Characteristics of the NAION cohort

**Fig. 1:**
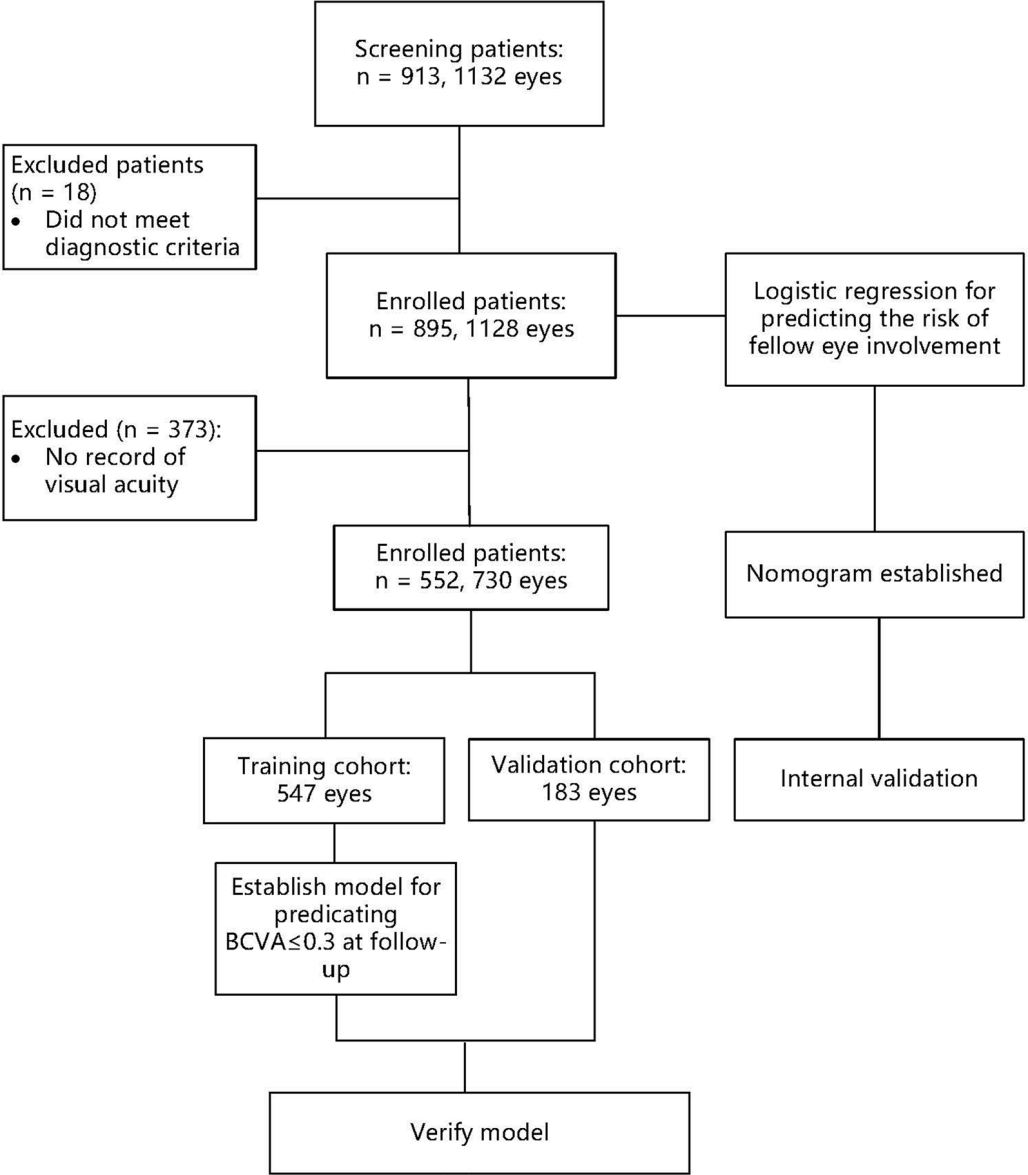
Study flowchart of patient disposition. BCVA, best-corrected visual acuity; n, number of patients

This study initially included 913 patients. After excluding 18 patients who did not meet the diagnostic criteria, 895 patients were included [12]. Among these patients, logistic regression was used to predict the risk factors for bilateral NAION involvement. We excluded patients without available BCVA records, and 522 patients (730 eyes) were enrolled to develop a model for predicting visual acuity progression. Of these patients, 392 (75%; 547 eyes) were included in the training cohort, and 130 patients (183 eyes) were included in the validation cohort (Fig. 1).

The demographic characteristics of the study population are summarized in Table 1. The mean age at onset of NAION was 55.6 ± 10.4 years, and 644 (71.96%) patients were aged > 50 years. Among the 895 patients, 560 (62.57%) were male. Bilateral NAION was present in 226 (25.3%) patients. Of these, 36 (4.0%) had simultaneous onset (second eye involvement within 7 days). The 5-year incidence of contralateral eye involvement was 14.5% (130/895). Risk factors for NAION development were hyperlipidemia (430/895, 48.04%), hypertension (331/895, 36.98%), diabetes mellitus (220/895, 24.58%), nocturnal arterial hypotension (284/395, 71.90%), OSAS (260/352, 73.86%), hematologic disorders (6/895, 0.67%), hyperhomocysteinemia (70/895, 7.82%), CAD or CVD (46/895, 5.14%), smoking history (223/895, 24.92%), alcohol consumption history (259/895, 28.94%), history of high-altitude living (10/895, 1.12%), and presence of a crowded optic disc (975/1128, 86.44%). Decreased visual acuity and visual field defects were the predominant clinical features of the initial manifestation of NAION. In this cohort, 34.0% (384/1128) of the affected eyes presented with decreased visual acuity, 29.3% (330/1128) exhibited visual field defects, and 33.4% (377/1128) concurrently manifested both symptoms during NAION onset. Additionally, 30.1% (340/1128) of the affected eyes showed peripapillary hemorrhage, and 6.7% (75/1128) exhibited macular serous detachment. The median BCVA at onset was 0.4 (IQR: 0.1–1.0). The follow-up visual acuity was obtained from 522 patients (730 eyes): a BCVA ≤ 0.1 was found in 219/730 eyes (30.0%), 0.1 < BCVA ≤ 0.3 in 150/730 eyes (20.6%), and a BCVA > 0.3 in 361/730 eyes (49.5%).

**Table 1:** Demographic and clinical features of patients with NAION

Variable	Total cohort (n = 895, 1,128 eyes)
Age (years); average (SD)	55.55 (10.37)
≥ 50 years; n (%)	644 (71.96%)
Sex, male; n (%)	560 (62.57%)
Bilateral eye involvement; n (%)	226 (25.25%)
Interval between two episodes (months); median (range)	5 (0–220)
Five-year incidence of contralateral eye involvement (%)	14.5% (130/895)
BMI (kg/m^2^); average (SD)	25.32 (3.21)
Risk factors; n (%)	
Hyperlipidemia	430 (48.04%)
Hypertension	331 (36.98%)
Diabetes mellitus	220 (24.58%)
Nocturnal hypotension	284/395 (71.90%)
OSAS	260/352 (73.86%)
Hematologic disorders	6 (0.67%)
Hyperhomocysteinemia	70 (7.82%)
CAD or CVD	46 (5.14%)
Smoking history	223 (24.92%)
Alcohol consumption history	259 (28.94%)
History of high-altitude living	10 (1.12%)
Ocular symptom at onset; number of eyes (%)	
Visual acuity loss	384 (34.04%)
Visual field defect	330 (29.26%)
Visual acuity loss and visual field defect	377 (33.42%)
Other	37 (3.28%)
Signs	
Crowded disc; number of eyes (%)	975 (86.44%)
Peripapillary hemorrhage; number of eyes (%)	340 (30.14%)
Macular serous detachment; number of eyes (%)	75 (6.65%)
BCVA (decimal) at onset; median (IQR)	0.4 (0.1–1.0)
BCVA (decimal) at follow-up; number of eyes (%)	
BCVA ≤ 0.1	219/730 (30.00%)
0.1 < BCVA ≤ 0.3	150/730 (20.55%)
BCVA > 0.3	361/730 (49.45%)

*BCVA* = best-corrected visual acuity; *BMI* = body mass index; *CAD* = coronary artery disease; *CVD* = cerebrovascular disease; *IQR* = interquartile range; *NAION* = non-arteritic anterior ischemic optic neuropathy; *OSAS* = obstructive sleep apnea syndrome; *SD* = standard deviation

The treatment was not standardized and was determined based on the clinical judgment of the treating neuro‑ophthalmologist. The choice of treatment was not randomized, and no comparative efficacy analysis was performed. The following interventions were recorded: (1) observation alone (i.e., no specific treatment) in 45.2% of patients; (2) intravenous methylprednisolone (80–120 mg) followed by oral tapering in 33.3% of patients; (3) oral prednisone (60–80 mg/day with gradual tapering over 3–4 weeks) in 21.5% of patients; and (4) risk factor modification (antihypertensives, lipid-lowering agents, and glycemic control) as per routine care.

### Factors associated with bilateral NAION

The baseline characteristics and risk factors of patients with NAION with unilateral versus bilateral involvement are presented in Table 2. Neither group differed significantly in terms of age (*P* = 0.15), sex (*P* = 0.06), and BMI (*P* = 0.36). Patients with bilateral NAION had a significantly higher prevalence of hyperlipidemia (*P* = 0.03), CAD or CVD (*P* < 0.05), smoking history (*P* < 0.01), and alcohol consumption history (*P* < 0.01) than those with unilateral NAION. As a corollary, the univariate logistic regression analysis revealed significant associations between bilateral NAION occurrence and female sex (OR = 1.38, 95% CI: 1.01–1.90, *P* < 0.05), history of hyperlipidemia (OR = 1.42, 95% CI: 1.05–1.93, *P* = 0.02), history of CAD or CVD (OR = 2.04, 95% CI: 1.04–3.88, *P* = 0.03), smoking history (OR = 2.24, 95% CI: 1.62–3.11, *P* < 0.01), and alcohol consumption history (OR = 2.03, 95% CI: 1.47–2.79, *P* < 0.01). In the multivariate analysis, hyperlipidemia (t = 2.02, *P* = 0.04), hematologic disorders (t = 2.31, *P* = 0.02), and smoking history (t = 4.79, *P* < 0.01) had a significant positive impact on the bilateral occurrence of NAION (Table 2).

**Table 2:** Risk factors of bilateral eye involvement in 895 patients with NAION

Variable	Patients with unilateral NAION (n = 669)	Patients with bilateral NAION (n = 226)	*P* value	Univariate analysis		Multivariate analysis		
				*P* value	OR (95% CI)	*P* value	t	B
Age (years); average (SD)	55.81 (10.91)	54.77 (8.89)	0.15	0.19	0.99 (0.98–1.00)			
Sex; n (%)								
Male	398 (59.8%)	152 (67.3%)	0.06	< 0.05	1.38 (1.01–1.90)			
Female	267 (40.2%)	74 (32.7%)		Reference				
BMI (kg/m^2^); average (SD)	25.29 (3.18)	25.52 (3.34)	0.36	0.35	1.02 (0.98–1.07)			
Risk factors; n (%)								
Hyperlipidemia	299 (45.4%)	122 (54.2%)	0.03*	0.02	1.42 (1.05–1.93)	0.04	2.02	0.06
Hypertension	232 (34.8%)	91 (40.3%)	0.17	0.14	1.26 (0.92–1.72)			
Diabetes mellitus	156 (23.4%)	57 (25.2%)	0.65	0.58	1.10 (0.77–1.56)			
Nocturnal hypotension	176 (69.0%)	104 (76.5%)	0.15	0.12	1.46 (0.91–2.37)			
OSAS	170 (73.6%)	87 (73.7%)	> 0.99	0.98	1.01 (0.61–1.68)			
Hematologic disorders	1 (0.2%)	3 (1.3%)	0.09	0.06	8.95 (1.14–181.36)	0.02	2.31	0.49
Hyperhomocysteinemia	58 (8.7%)	12 (5.3%)	0.13	0.10	0.59 (0.30–1.08)			
CAD or CVD	24 (3.6%)	16 (7.1%)	< 0.05*	0.03	2.04 (1.04–3.88)			
Smoking history	141 (21.2%)	78 (34.5%)	< 0.01*	< 0.01	2.24 (1.62–3.11)	< 0.01	4.79	0.16
Alcohol consumption history	166 (24.9%)	88 (38.9%)	< 0.01*	< 0.01	2.03 (1.47–2.79)			
History of high-altitude living	4 (0.6%)	2 (0.9%)	> 0.99	0.65	1.48 (0.20–7.66)			

*BMI* = body mass index; *CAD* = coronary artery disease; *CI* = confidence interval; *CVD* = cerebrovascular disease; *NAION* = non-arteritic anterior ischemic optic neuropathy; *OR* = odds ratio; *OSAS* = obstructive sleep apnea syndrome; *SD* = standard deviation

^*^Pearson's chi-squared test

### Development and assessment of the model for predicting BCVA ≤ 0.3

Among 522 eligible patients, 392 were randomly assigned to the training cohort. The mean age was 53.6 ± 9.7 years, and 354 (66.5%) patients were male. The validation cohort comprised 130 patients. The baseline data did not differ significantly between the training and validation cohorts (Table 3).

**Table 3:** Baseline characteristics of 522 patients with follow-up data in the training and validation cohorts

Variable	Training cohort (n = 392, 547 eyes)	Validation cohort (n = 130, 183 eyes)	*P* value
Age (years); average (SD)	53.56 (9.74)	53.49 (8.92)	0.93
Sex; n (%)			0.74
Male	364 (66.54%)	118 (64.84%)	
Female	183 (33.46%)	64 (35.16%)	
BMI (kg/m^2^); average (SD)	25.54 (3.34)	25.89 (3.30)	0.23
Risk factors; n (%)			
Hyperlipidemia			0.09
Yes	273 (50.65%)	105 (58.33%)	
No	266 (49.35%)	75 (41.67%)	
Hypertension			0.36
Yes	182 (33.27%)	68 (37.36%)	
No	365 (66.73%)	114 (62.64%)	
Diabetes mellitus			0.14
Yes	146 (26.69%)	38 (20.88%)	
No	401 (73.31%)	144 (79.12%)	
Nocturnal hypotension			0.67
Yes	272 (71.39%)	96 (73.85%)	
No	109 (28.61%)	34 (26.15%)	
OSAS			0.12
Yes	223 (69.47%)	87 (77.68%)	
No	98 (30.53%)	25 (22.32%)	
Hematologic disorders			0.83
Yes	6 (1.10%)	1 (0.55%)	
No	541 (98.90%)	181 (99.45%)	
Hyperhomocysteinemia			0.92
Yes	30 (5.48%)	11 (6.04%)	
No	517 (94.52%)	171 (93.96%)	
CAD or CVD			0.87
Yes	41 (7.50%)	15 (8.24%)	
No	506 (92.50%)	167 (91.76%)	
Smoking history			0.10
Yes	169 (30.90%)	69 (37.91%)	
No	378 (69.10%)	113 (62.09%)	
Alcohol consumption history			0.42
Yes	202 (36.93%)	74 (40.66%)	
No	345 (63.07%)	108 (59.34%)	
History of high-altitude living			0.22
Yes	8 (1.46%)	0 (0.00%)	
No	539 (98.54%)	183 (100.00%)	
Symptoms; number of eyes (%)			
Crowded disc			0.20
Yes	274 (51.99%)	102 (57.95%)	
No	253 (48.01%)	74 (42.05%)	
Peripapillary hemorrhage			0.07
Yes	165 (33.20%)	41 (25.15%)	
No	332 (66.80%)	122 (74.85%)	
Macular serous detachment			0.55
Yes	31 (6.31%)	13 (8.07%)	
No	460 (93.69%)	148 (91.93%)	

*BMI* = body mass index; *CAD* = coronary artery disease; *CVD* = cerebrovascular disease; *OSAS* = obstructive sleep apnea syndrome; *SD* = standard deviation

*P* values were determined using the Student's t-test and Pearson's chi-squared test

**Fig. 2:**
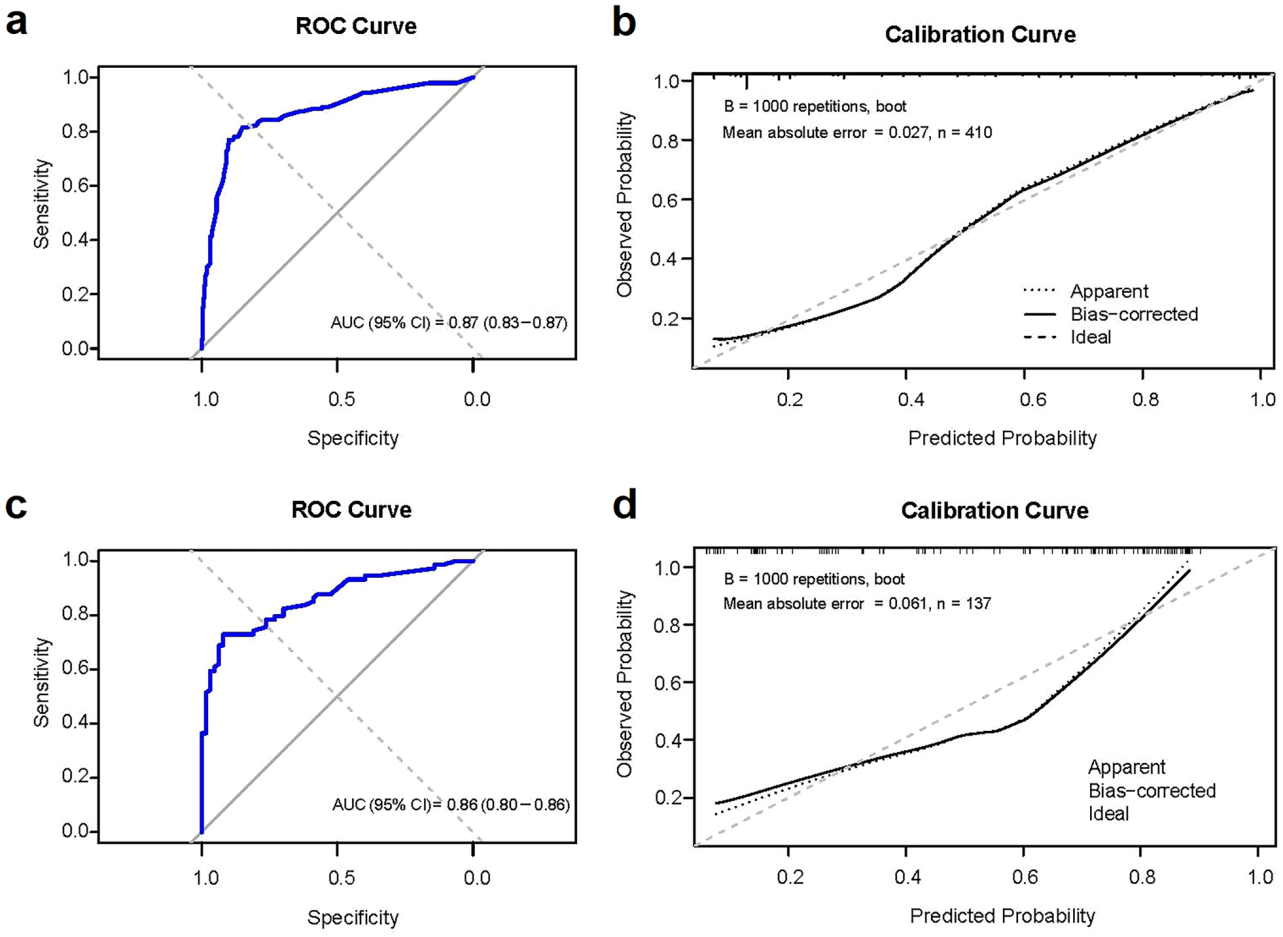
Receiver operating characterstic (ROC) and calibration curves for predictive value of the model for factors associated with BCVA ≤ 0.3. **a** The AUC in the training cohort was 0.87 (95% CI: 0.83–0.87); **b** The calibration curve for the training cohort; **c** The AUC for the validation cohort was 0.86 (95% CI: 0.80–0.86); **d** The calibration curve for the validation cohort. AUC, area under the curve; BCVA, best-corrected visual acuity; confidence interval

To identify factors associated with visual acuity at follow-up, all included variables were preliminarily screened using LASSO regression analysis. Variables with non-zero coefficients from the LASSO regression results were included in the multivariate logistic regression analysis. In the training cohort, BCVA ≤ 0.3 at follow-up was associated with a lower BCVA at onset (OR = 0.01, 95% CI: 0.00–0.04, *P* < 0.01) and a higher prevalence of diabetes mellitus (OR = 2.99, 95% CI: 1.20–7.88, *P* = 0.02), whereas a higher prevalence of peripapillary hemorrhages was associated with a higher prevalence of visual recovery (OR = 0.23, 95% CI: 0.08–0.59, *P* < 0.01). After adjusting for within-patient correlations using cluster-robust standard errors, a lower baseline BCVA (OR = 0.72, 95% CI: 0.64–0.82, *P* < 0.01), diabetes (OR = 2.97, 95% CI: 1.10–8.03, *P* = 0.03), hyperhomocysteinemia (OR = 11.59, 95% CI: 1.87–71.97, *P* = 0.01), and macular serous detachment (OR = 6.70, 95% CI: 1.34–33.40, *P* = 0.02) were independently associated with poor visual outcomes (BCVA ≤ 0.3). Peripapillary hemorrhage was a protective factor (OR = 0.19, 95% CI: 0.06–0.66, *P* < 0.01) (Table 4). Other variables were not statistically significant. The AUC values for the training and validation cohorts were 0.87 (95% CI: 0.83–0.87) and 0.86 (95% CI: 0.80–0.86), respectively (Fig. 2a, b). The calibration curve showed that the actual predicted lines for the training and validation cohorts closely overlapped the ideal predicted lines (Fig. 2c, d).

**Table 4:** Characteristics of the training cohort screened by LASSO and multivariate logistic regression analyses

Variables	BCVA ≤ 0.3 at follow-up (259 eyes)	BCVA > 0.3 at follow-up (282 eyes)	LASSO regression	Logistic regression		
			Coefficient	Β (SE)	*P* value	OR (95% CI)
Age (years); average (SD)	53.63 (9.72)	53.56 (9.73)	0.004	0.15	0.88	1.00 (0.95–1.06)
Sex; n (%)						
Male	175 (67.57%)	186 (65.96%)	− 0.42	− 1.71	0.09	0.33 (0.09–1.17)
Female	84 (32.43%)	96 (34.04%)	Reference			
BCVA at onset; median (IQR)	0.22 (0.29)	0.71 (0.32)	− 3.38	− 5.08	< 0.01*	0.72 (0.64–0.82)
Risk factors; n (%)						
Hyperlipidemia						
Yes	132 (51.97%)	139 (49.64%)	0.18	1.68	0.09	2.10 (0.88–5.03)
No	122 (48.03%)	141 (50.36%)	Reference			
Hypertension						
Yes	99 (38.22%)	80 (28.37%)	0.03	0.05	0.96	1.02 (0.40–2.64)
No	160 (61.78%)	202 (71.63%)	Reference			
Diabetes mellitus						
Yes	72 (27.80%)	72 (25.53%)	0.56	2.14	0.03*	2.97 (1.10–8.03)
No	187 (72.20%)	210 (74.47%)	Reference			
Nocturnal hypotension						
Yes	130 (73.03%)	139 (69.85%)	− 0.38	− 1.53	0.13	0.47 (0.17–1.24)
No	48 (26.97%)	60 (30.15%)	Reference			
OSAS						
Yes	115 (70.99%)	106 (67.52%)	0.09	1.16	0.24	1.92 (0.64–5.79)
No	47 (29.01%)	51 (32.48%)	Reference			
Hyperhomocysteinemia						
Yes	16 (6.18%)	14 (4.96%)	0.50	2.63	0.01*	11.59 (1.87–71.97)
No	243 (93.82%)	268 (95.04%)	Reference			
CAD or CVD						
Yes	24 (9.27%)	17 (6.03%)	0.02	0.27	0.79	1.17 (0.38–3.63)
No	235 (90.73%)	265 (93.97%)	Reference			
Smoking history						
Yes	83 (32.05%)	85 (30.14%)	− 0.20	− 1.18	0.24	0.56 (0.22–1.46)
No	176 (67.95%)	197 (69.86%)	Reference			
Symptoms, n (%)						
Peripapillary hemorrhage						
Yes	73 (31.33%)	91 (34.73%)	− 0.76	− 2.60	0.01*	0.19 (0.06–0.66)
No	160 (68.67%)	171 (65.27%)	Reference			
Macular serous detachment						
Yes	19 (8.23%)	12 (4.65%)	0.82	2.32	0.02*	6.70 (1.34–33.40)
No	212 (91.77%)	246 (95.35%)	Reference			

*BCVA* = best-corrected visual acuity; *BMI *= body mass index; *CAD* = coronary artery disease; *CI* = confidence interval; *CVD* = cerebrovascular disease; *IQR* = interquartile range; *OR* = odds ratio; *OSAS* = obstructive sleep apnea syndrome; *SD* = standard deviation

*P* values were determined using the multivariate logistic regression analyses; *P < 0.05

## Discussion

This study retrospectively summarized the clinical baseline characteristics and prognosis of a large cohort of Chinese patients with NAION from various clinical centers participating in the Chinese Neuro-Ophthalmic Diseases Project.

NAION usually occurs in patients aged over 50 years, whereas patients younger than 50 years can also be affected [13]. In our study cohort, approximately 30% of patients were diagnosed with NAION at an age younger than 50 years. Preechawat et al. assessed the data of 727 patients with NAION and found that 169 (23.2%) were aged < 50 years [14]. In another study, 43 (7.5%) of 577 patients with NAION were aged < 50 years [15]. Patients with NAION occurring at < 50 years of age may have a different vasculopathic risk factor profile. One study indicated that the prevalence of hyperlipidemia and diabetes is significantly higher in younger patients with NAION than in those aged > 50 years [15].

Optic disc drusen (ODD) was more prevalent in the younger group with NAION than was expected according to population-based studies (51% versus 2%, respectively) [16]. Although earlier studies have reported no sex differences [12], our study found a male-to-female ratio of approximately 3:2 in our cohort of patients with NAION. A recent large-scale meta-analysis reported a correlation between male sex and higher NAION prevalence, particularly in Asian populations [17, 18 ]. This concurs with the findings from our study, in which male sex increased the individual risk of developing NAION by 32%–36% [19, 20 ]. NAION has been associated with different combinations of local and systemic risk factors [21–24]. The most frequent systemic comorbidities are arterial hypertension (34%–49%), diabetes mellitus (5%–25%), and hyperlipidemia (53%) [25, 26 ]. Our results agreed with published clinical features of patients with NAION. Approximately 48% of patients with NAION had concomitant hyperlipidemia, 37% had hypertension, and 25% had diabetes.

When analyzing the symptoms at the onset of NAION, we found that more than 85% of the affected eyes displayed characteristics of a crowded disc, approximately 30% presented with concurrent peripapillary hemorrhage at onset, and 6% exhibited a macular serous detachment. A crowded disc has been found in the affected or contralateral eye in 80%–90% of patients with NAION [4, 27 ]. A crowded optic disc or pseudopapilledema is the surrogate of a small or mini optic disc. Peripapillary hemorrhages were frequently observed; however, a recent case series reported that 50% of patients with NAION have peripapillary hemorrhages, and a previous study reported that approximately 20% of patients have macular serous detachment [28, 29 ], which was not comparable to our study findings. However, previous studies have been limited by relatively small sample sizes.

The reported incidence of contralateral eye involvement varies from 24% to 39%, and inter-study differences in follow-up durations may explain the relatively wide range [5, 30 ]. A similar observation was made regarding the contralateral eye involvement in our cohort. Patients with either simultaneous or sequential bilateral NAION involvement accounted for 25% of the study population. Simultaneous bilateral NAION (36/226, 15.9%) may have resulted from an acute systemic insult affecting both optic nerves simultaneously, such as severe nocturnal hypotension, profound anemia, or acute blood loss. In contrast, sequential bilateral NAION (190/226, 84.1%) likely reflects a chronic anatomical predisposition (e.g., bilateral crowded discs) combined with ongoing vascular risk factors (e.g., hyperlipidemia and smoking) that increase the risk over time. Due to the small number of simultaneous cases, a separate risk factor analysis was not feasible. Future studies with larger cohorts are required to validate these mechanisms.

Studies have shown that diabetes, bilateral ODD, and OSAS are risk factors for the involvement of the fellow eye [31, 32 ]. Multivariate regression analysis in our study revealed that smoking was a significant risk factor for secondary eye involvement. The significant association between smoking and CVD has been well documented [33]. While some studies have not found such a relationship [34], Chung et al. reported that smokers develop NAION at a significantly younger age than non-smokers [35]. This study also found that hyperlipidemia and hematologic disorders were significantly associated with contralateral eye involvement. Studies have identified diabetes, anemia, and chronic renal failure as potential risk factors for contralateral eye involvement. We consider this conclusion reasonable because systemic alterations in the blood state can lead to changes in the blood supply to various organs, including the quality and quantity of blood flow in the bilateral short posterior ciliary arteries. These changes, combined with the synergistic effects of other risk factors, may contribute to the development of bilateral NAION.

Regarding the prognosis of NAION patients, we found that nearly 50% of patients had a final BCVA of 0.3 or better, while approximately 30% had a BCVA < 0.1. Previous studies have reported that the final visual acuity of patients with untreated NAION is highly variable, with 50% of patients reaching a BCVA ≥ 20/30 and 25% of patients achieving a BCVA ≤ 1/10 [36]. Additionally, multivariate regression analyses revealed that factors such as the severity of the vision loss at onset, diabetes mellitus, hyperhomocysteinemia, and macular serous detachment were predictors of a worse visual prognosis after a diagnosis of NAION. The severity of vision loss at onset and diabetes mellitus have also been significantly associated with poor visual improvement [10, 32 ]. Hyperhomocysteinemia may worsen visual outcomes by inducing oxidative stress, endothelial dysfunction, and microthrombosis, thereby aggravating optic nerve head ischemia [37, 38 ]. However, a secondary case-control analysis of the multicenter, prospective, randomized controlled trial for NAION therapy found that diabetes mellitus, hypertension, hyperlipidemia, and cardiovascular risk factors did not increase the risk of progression [39, 40 ]. This conclusion is based on a study of patients with acute NAION, assessed from screening to day 1, which primarily evaluated short-term changes in visual acuity. In contrast, our study focused on evaluating the visual prognosis of patients with NAION during the stable phase. Macular serous detachment, a marker of severe disc edema, has shown a trend toward a poorer prognosis, possibly due to reversible fluid accumulation in some patients, whereas persistent detachment may damage photoreceptors [41, 42 ]. In our study, peripapillary hemorrhage at baseline was independently associated with better visual recovery. We speculate that peripapillary hemorrhage in acute NAION likely reflects early, reversible disc edema rather than irreversible axonal damage. Patients with hemorrhage presented earlier (within 7 days) and thus had a greater potential for spontaneous visual improvement.

This study had some limitations. First, this study was a retrospective analysis and lacked prospective longitudinal follow-up data. Several studies have indicated that peripapillary and macular microvasculature features of NAION support the role of OCT angiography (OCTA) metrics in assessing disease severity and their demonstration of progressive vessel density reduction from acute to chronic NAION [43, 44]. However, optic nerve head OCT/OCTA parameters (e.g., peripapillary retinal nerve fiber layer thickness and disc perfusion) were not available for this cohort because the imaging protocol targeted the macula. Moreover, ODD is a known anatomical risk factor for NAION. However, there was no systematic evaluation of ODD, as many patients lacked data from enhanced depth imaging OCT for a clear diagnosis. Future prospective studies should include comprehensive optic nerve head imaging to assess the independent contribution of ODD to visual prognosis. Second, nocturnal hypotension was not included in the final prediction model because ABPM data were available for only 44.1% of the patients, and excluding those with missing data would have substantially reduced the sample size and introduced selection bias. Third, our classification of unilateral NAION was based on the last available follow-up without considering censoring or varied follow-up durations, which could introduce time-dependent biases. Future prospective studies with fixed follow-up intervals or survival analyses are required to validate our findings. Although this is the largest cohort of patients with NAION in China to date, the generalizability of the results to other populations has not been established. In particular, a small optic disc is a major risk factor for NAION, and population-based studies have shown that Chinese individuals have a larger mean optic disc size than Caucasians. A strength of our investigation was that it involved the first and largest nationwide cohort of patients with NAION in China for the analysis of the risk of bilateral involvement and visual prognosis.

## Conclusions

In conclusion, based on this relatively large cohort, we found that hyperlipidemia, hematologic disorders, and smoking were significant risk factors for bilateral NAION. In addition, the severity of vision loss at onset, diabetes mellitus, hyperhomocysteinemia, and macular serous detachment were associated with a poor visual prognosis.

## Data Availability

The datasets generated and/or analyzed during the current study are not publicly available owing to data management requirements but are available from the corresponding author upon reasonable request.

## Abbreviations

ABPM: Ambulatory blood pressure monitoring
AUC: Area under the curve
BCVA: Best-corrected visual acuity
BMI: Body mass index
CAD: Coronary artery disease
CI: Confidence interval
CT: Computed tomography
CVD: Cerebrovascular disease
FDG-PET: (18)F-fluorodeoxyglucose positron emission tomography
IQR: Interquartile range
LASSO: Least absolute shrinkage and selection operator
NAION: Non-arteritic anterior ischemic optic neuropathy
OCT: Optical coherence tomography
OCTA: Optical coherence tomography angiography
ODD: Optic disc drusen
OR: Odds ratio
OSAS: Obstructive sleep apnea syndrome
PDE-5: Phosphodiesterase-5
ROC: Receiver operating characteristic
